# Bonnesen-style inequalities on surfaces of constant curvature

**DOI:** 10.1186/s13660-018-1918-1

**Published:** 2018-11-23

**Authors:** Min Chang

**Affiliations:** School of Mathematics and Statistics, Southwest University, Chongqing, People’s Republic of China

**Keywords:** 52A22, Isoperimetric inequality, Bonnesen-style inequality, Isoperimetric deficit, Surface of constant curvature

## Abstract

In this paper, some Bonnesen-style inequalities on a surface $\mathbb {X}_{\kappa}$ of constant curvature *κ* (i.e., the Euclidean plane $\mathbb{R}^{2}$, projective plane $\mathbb{R}P^{2}$, or hyperbolic plane $\mathbb{H}^{2}$) are proved. The method is integral geometric and gives a uniform proof of some Bonnesen-style inequalities alone with equality conditions.

## Introduction

The classical isoperimetric problem dates back to antique literature and geometry. The problem can be stated as: Among all closed curves of given length in the Euclidean plane $\mathbb {R}^{2}$, which one maximizes the area of its enclosed region?

The solution to the problem is usually expressed in the form of an inequality that relates the length $P_{K}$ of a rectifiable simple closed curve and the area $A_{K}$ of the planar region *K* that the curve encloses in $\mathbb{R}^{2}$. The solution to the classical isoperimetric problem is characterized as the following isoperimetric inequality:

1.1 $$ P_{K}^{2}-4\pi A_{K}\geq0, $$

with equality if and only if *K* is a Euclidean disc.

The history of geometric proofs for the classical isoperimetric problem goes back to Ancient Greeks and was recorded by Pappus of Alexandria in the fourth century AD, but their arguments were incomplete. The first progress towards the solution was made by Steiner [22] in 1838 by a geometric method later named Steiner symmetrization. His proof contained a flaw that later was fixed by analytic approach. In 1870, Weierstrass gave the first rigorous proof as a corollary of his theory of calculus of several variables. Since then, many other proofs have been discovered. In 1902, Hurwitz [10] published a short proof using the Fourier series that applies to arbitrary rectifiable curves (not assumed to be smooth). An elegant direct proof based on comparison of a simple closed curve of length *L* with the circle of radius $\frac{L}{2\pi}$ was given by Schmidt [20]. See [4, 15, 16, 21] for more references.

In 1920s, Bonnesen proved a series of inequalities of the form [3]

1.2 $$ P_{K}^{2}-4\pi A_{K}\geq B_{K}, $$

where $B_{K}$ is a non-negative invariant and vanishes if and only if the domain *K* is a Euclidean disc.

A well-known Bonnesen-style inequality is

1.3 $$ P_{K}^{2}-4\pi A_{K}\geq \pi^{2}(R_{{K}}-r_{K})^{{2}}, $$

where $R_{K}$ and $r_{K}$ respectively denote the circumradius and inradius of *K*, with equality if and only if *K* is a Euclidean disc.

Many inequalities of style (1.2), called Bonnesen-style inequalities, were found along with variations and generalizations in the past decades [2, 5, 6, 8, 11, 19, 29–34]. On the other hand, the classical isoperimetric inequality has been extended to higher dimensions and a surface of constant curvature *κ*, i.e., the Euclidean plane $\mathbb{R}^{2}$, projective plane $\mathbb{R}P^{2}$, or hyperbolic plane $\mathbb{H}^{2}$.

Let *K* be a compact set bounded by a rectifiable simple closed curve with the area $A_{K}$ and perimeter $P_{K}$ in $\mathbb{X}_{\kappa}$. Then [1, 7, 9, 14, 17, 18, 23–26]

1.4 $$ P_{K}^{2}-(4\pi-\kappa A_{K})A_{K} \geq0, $$

with equality if and only if *K* is a geodesic disc.

The geodesic disc of radius *r* with center *x* is defined as

$$B_{\kappa}(x, r)=\bigl\{ y\in\mathbb{X}_{\kappa}: d(x,y)\leq r\bigr\} , $$

where *d* is the geodesic distance function in $\mathbb{X}_{\kappa}$. The area, perimeter of $B_{\kappa}(x,r)$ in $\mathbb{X}_{\kappa}$ are respectively [12]

1.5 $$ A\bigl(B_{\kappa}(x,r)\bigr)=\frac{2\pi}{\kappa}\bigl(1- \operatorname{cn}_{\kappa}(r)\bigr), \qquad P\bigl(B_{\kappa}(x,r) \bigr)=2\pi\operatorname{sn}_{\kappa}(r). $$

The limiting cases of as $\kappa\rightarrow0$ yield the Euclidean formulas $A(B(x,r))=\pi r^{2}$ and $P(B(x,r))=2\pi r$.

A Bonnesen-type inequality in $\mathbb{X}_{\kappa}$ is of the form

1.6 $$ P^{2}_{K}-(4\pi-\kappa A_{K})A_{K} \geq B_{K}, $$

where $B_{K}$ vanishes if and only if *K* is a geodesic disc [15, 28].

Bonnesen [3] established an inequality of the type (1.6) in the sphere of radius $1/\sqrt{\kappa}$:

1.7 $$ P^{2}_{K}-(4\pi-\kappa A_{K})A_{K} \geq4\pi^{2}\ \operatorname{tn}_{\kappa}^{2} \biggl( \frac{R_{K}-r_{K}}{2} \biggr), $$

where $R_{K}$ and $r_{K}$ are respectively the minimum circumscribed radius and the maximum inscribed radius of *K*.

Let $\mathbb{X}_{\kappa}$ be the surface of constant curvature *κ*, specifically:

$$ \mathbb{X}_{\kappa}= \textstyle\begin{cases} \mathbb{P}R^{2}, \text{Euclidean 2-sphere of radius } 1/\sqrt{\kappa}, & \text{if } \kappa>0;\\ \mathbb{R}^{2}, \text{Euclidean plane}, &\text{if } \kappa=0;\\ \mathbb{H}^{2}, \text{Hyperbolic plane of constant curvature } \kappa, & \text{if } \kappa< 0. \end{cases} $$

Let

1.8 $$ \Delta_{\kappa}(K) =P^{2}_{K}-(4\pi- \kappa A_{K})A_{K} $$

denote the isoperimetric deficit of *K* in $\mathbb{X}_{\kappa}$. The trigonometric functions appearing in (1.7) are defined by

$$\begin{gathered} \operatorname{sn}_{\kappa}(t) = \textstyle\begin{cases} \frac{1}{\sqrt{-\kappa}} \sinh(\sqrt{-\kappa} t), &\kappa< 0,\\ t, & \kappa=0,\\ \frac{1}{\sqrt{\kappa}} \sin(\sqrt{\kappa} t),& \kappa>0; \end{cases}\displaystyle \\ \operatorname{cn}_{\kappa}(t)= \textstyle\begin{cases} \cosh(\sqrt{-\kappa}t), &\kappa< 0,\\ 1, &\kappa=0,\\ \cos(\sqrt{\kappa}t), &\kappa>0; \end{cases}\displaystyle \\\operatorname{tn}_{\kappa}(t)= \frac{\operatorname{sn}_{\kappa}(t)}{\operatorname{cn}_{\kappa}(t)}; \qquad \operatorname{ct}_{\kappa}(t)= \frac{\operatorname{cn}_{\kappa}(t)}{\operatorname{sn}_{\kappa}(t)};\end{gathered} $$

and

1.9 $$ \textstyle\begin{array}{lll} {\kappa}\cdot\operatorname{sn}^{2}_{\kappa}(t)+ \operatorname{cn}^{2}_{\kappa}(t)=1. \end{array} $$

The following Bonnesen-type inequality is obtained in [31]:

1.10 $$ \Delta_{\kappa}(K) \geq \biggl(2\pi-\frac{\kappa}{2} A_{K} \biggr)^{2} \biggl(\operatorname{tn}_{\kappa} \frac{R_{K}}{2} -\operatorname{tn}_{\kappa}\frac{r_{K}}{2} \biggr)^{2} $$

for a convex set *K*, with equality if *K* is a geodesic disc.

Inequality (1.10) was strengthened [28] as

1.11 $$ \begin{aligned}[b] \Delta_{\kappa}(K) \geq{}& \biggl(2\pi-\frac{\kappa}{2} A_{K} \biggr)^{2} \biggl(\operatorname{tn}_{\kappa}\frac{R_{K}}{2} -\operatorname{tn}_{\kappa}\frac{r_{K}}{2} \biggr)^{2}\\ & + \biggl(2\pi-\frac{\kappa}{2} A_{K} \biggr)^{2} \biggl(\operatorname{tn}_{\kappa}\frac{R_{K}}{2} +\operatorname{tn}_{\kappa}\frac{r_{K}}{2} -\frac{2P_{K}}{4\pi-\kappa A_{K}} \biggr)^{2}, \end{aligned} $$

with equality if *K* is a geodesic disc.

For a convex set *K* in $\mathbb{X}_{\kappa}$ such that $(2\pi-\kappa A_{K})^{2}+\kappa P_{K}^{2}\geq0$ if $\kappa<0$, Klain [12] obtained the following Bonnesen-style inequality:

1.12 $$ \Delta_{\kappa}(K) \geq \frac{ ( (2\pi-\kappa A_{K} )^{2} +\kappa P_{K}^{2} )^{2}}{ 4(2\pi-\kappa A_{K})^{2}} \bigl( \operatorname{sn}_{\kappa}(R_{K})-\operatorname{sn}_{\kappa }(r_{K}) \bigr)^{2}, $$

with equality if *K* is a geodesic disc.

For more results on Bonnesen-style inequality, see, e.g., [1, 2, 5–9, 11, 14, 17, 19, 23–26, 29–34].

The purpose of this paper is to find a new Bonnesen-style inequality with equality condition on surfaces $\mathbb{X}_{\kappa}$ of constant curvature, especially on the hyperbolic plane $\mathbb{H}^{2}$ by integral geometric method. We are going to seek the following Bonnesen-style inequality for a convex set *K* in $\mathbb{X}_{\kappa}$:

$$ \Delta_{\kappa}(K) \geq \pi^{2} \bigl(\operatorname{tn}_{\kappa}(R_{K})- \operatorname{tn}_{\kappa }(r_{K}) \bigr)^{2}, $$

with equality if and only if *K* is a hyperbolic disc.

Finally, we give some special cases of these Bonnesen-style inequalities that strengthen some known Bonnesen-style inequalities in the Euclidean plane including the Bonnesen isoperimetric inequality (1.3).

## Preliminaries

Let $\mathcal{C}(\mathbb{X}_{\kappa})$ be the set of all convex sets with perimeter $P_{K}\leq \frac{2\pi}{\sqrt{\kappa}}$ if $\kappa>0$ in $\mathbb{X}_{\kappa}$. For a fixed point $x_{0}\in\mathbb{X}_{\kappa}$, the geodesic disc of radius *r* with center $x_{0}$ is the set of points that lie at most a distance *r* from $x_{0}$ in $\mathbb{X}_{\kappa}$. For $K\in\mathbb{X}_{\kappa}$, let $A_{K}$ and $P_{K}$ denote the area and the perimeter of *K*, respectively. Let $r_{K}$ and $R_{K}$ be the maximum inscribed radius and the minimum circumscribed radius of *K*, respectively. We always assume that *K* lies in an open hemisphere of $\mathbb {P}R^{2}$ such that $R_{K}<\frac{\pi}{2\sqrt{\kappa}}$.

A set *K* is convex if, for points $x, y\in K$, the shortest geodesic curve connecting *x*, *y* belongs to *K*. It should be noted that, for a convex set *K* in $\mathbb{P}R^{2}$, $2\pi-\kappa A_{K}>0$.

Let $G_{\kappa}$ be the group of isometries in $\mathbb {X}_{\kappa}$, and let *dg* be the kinematic density (Harr measure) on $G_{\kappa}$. Let *K* be fixed and *gL* as moving via the isometry $g\in G_{\kappa}$. For $K, L\in\mathbb{X}_{\kappa}$, let $\chi(K\cap gL)$ and $\sharp(\partial K\cap \partial(gL))$ be the Euler–Poincaré characteristic of $K\cap gL$ and the number of points of the intersection $\partial K\cap\partial (gL)$, respectively.

The following fundamental kinematic formula is due to Blaschke [19]:

2.1 $$ \int_{\{g: K\cap gL\ne\emptyset\}} \chi(K\cap gL) \,dg =2\pi(A_{K}+A_{L})+P_{K}P_{L}- \kappa A_{K}A_{L}. $$

As the limiting case, when *K*, *L* degenerate to curves *∂K*, *∂L*, respectively, then $A_{K}=A_{L}=0$ and the perimeters are $2P_{K}$, $2P_{L}$. Hence we have the following kinematic formula of Poincaré [19]:

2.2 $$ \int_{\{g: \partial K\cap\partial(gL)\ne\emptyset\}} \sharp \bigl(\partial K\cap \partial(gL)\bigr) \,dg =4P_{K}P_{L}. $$

Since the compact sets are assumed to be simply connected and enclosed by simple curves, $\chi (K\cap gL)=n(g)\equiv (\text{the number of connected components of the intersection }K\cap gL)$. Let $\mu=\{g \in G_{\kappa}: K\subset gL \text{ or } K\supset gL \}$, then the fundamental kinematic formula of Blaschke (2.1) can be rewritten as [31]:

2.3 $$ \int_{\mu}dg + \int_{\{g: \partial K\cap\partial(gL)\ne\emptyset\}} n(g) \,dg =2\pi(A_{K}+A_{L})+P_{K}P_{L}- \kappa A_{K}A_{L}. $$

When $\partial K\cap\partial(gL)\ne\emptyset$, each component of $K\cap gL$ is bounded by at least an arc of *∂K* and an arc of $\partial(g L)$, and $n(g)\le\sharp(\partial K\cap\partial(gL))/2$. Then the following containment measure inequality is an immediate consequence of Poincaré ’s formula (2.2) and Blaschke’s formula (2.3) [12, 13, 19].

### Lemma 2.1

*Let* *K*, *L* *be two compact sets bounded by rectifiable simple closed curves in* $\mathbb {X}_{\kappa}$, *then*

2.4 $$ \int_{\mu}dg \geq2\pi(A_{K}+A_{L})-P_{K}P_{L}- \kappa A_{K}A_{L}. $$

If $K\equiv L$, then there is no $g\in G_{\kappa}$ such that $gK\supset K$ nor $gK\subset K$. Hence $\int_{\mu}dg=0$ and inequality (2.4) immediately leads to the isoperimetric inequality (1.2).

The following Bonnesen inequality in $\mathbb {X}_{\kappa}$ is important for our main results [27].

### Lemma 2.2

*For* $K\in\mathcal{C}(\mathbb{X}_{\kappa})$, *let* $R_{K}$ *and* $r_{K}$ *be respectively the maximum inscribed radius and the minimum circumscribed radius of* *K*. *Then*, *for* $r_{K}\leq r \leq R_{K}$,

2.5 $$ \bigl[ (2\pi-\kappa A_{K} )^{2}+\kappa P_{K}^{2} \bigr] \operatorname{sn}^{2}_{\kappa}(r) -4\pi P_{K} \operatorname{sn}_{\kappa}(r) -A_{K}( \kappa A_{K}-4\pi) \leq0. $$

### Proof

Let *L* be a geodesic disc of radius *r*. Then neither $gB_{\kappa}(r)\subset K$ nor $gB_{\kappa}(r)\supset K$ for any $g\in G_{\kappa}$ and hence the measure $\int_{\mu}dg =0$. By (1.5) and (2.4) we have

2.6 $$ P_{K} \operatorname{sn}_{\kappa}(r) - \biggl( \frac{2\pi}{\kappa}-A_{K} \biggr) \bigl(1-\operatorname{cn}_{\kappa}(r) \bigr)-A_{K} \geq0. $$

Identity (1.9) shows $1-{\kappa}\cdot\operatorname{sn}^{2}_{\kappa}(r)= \operatorname{cn}^{2}_{\kappa}(r)>0$ and inequality (2.6) can be rewritten as

2.7 $$ P_{K} \operatorname{sn}_{\kappa}(r)- \frac{2\pi}{\kappa} \geq \biggl(A_{K}-\frac{2\pi}{\kappa} \biggr) \sqrt {1-{\kappa}\cdot\operatorname{sn}^{2}_{\kappa}(r)}. $$

For $\kappa\geq0$,

$$ P_{K} \operatorname{sn}_{\kappa}(r)- \frac{2\pi}{\kappa}\leq0, $$

hence by squaring both sides of (2.7) we have

$$ \biggl(P_{K} \operatorname{sn}_{\kappa}(r)-\frac{2\pi}{\kappa} \biggr)^{2} \leq \biggl(A_{K}-\frac{2\pi}{\kappa} \biggr)^{2} \bigl(1-{\kappa}\cdot\operatorname{sn}^{2}_{\kappa}(r) \bigr), $$

that is,

$$ \bigl( (2\pi-\kappa A_{K} )^{2}+\kappa P_{K}^{2} \bigr) \operatorname{sn}^{2}_{\kappa}(r) -4\pi P_{K} \operatorname{sn}_{\kappa}(r) -A_{K}(\kappa A_{K}-4 \pi) \leq 0. $$

For $\kappa<0$, then $A_{K}-\frac{2\pi}{\kappa}>0$. Squaring both sides of (2.7) leads to

$$ \biggl(P_{K} \operatorname{sn}_{\kappa}(r)-\frac{2\pi}{\kappa} \biggr)^{2} \geq \biggl(A_{K}-\frac{2\pi}{\kappa} \biggr)^{2} \bigl(1-{\kappa}\cdot\operatorname{sn}^{2}_{\kappa}(r) \bigr), $$

i.e.,

$$ \bigl( (2\pi-\kappa A_{K} )^{2}+\kappa P_{K}^{2} \bigr) \operatorname{sn}^{2}_{\kappa}(r) -4\pi P_{K} \operatorname{sn}_{\kappa}(r) -A_{K}(\kappa A_{K}-4 \pi) \leq0. $$

 □

We are now in the position to prove our Bonnesen-style inequalities.

### Theorem 2.1

*Let* $K\in\mathcal{C}(\mathbb{X}_{\kappa})$. *If* $r_{K}\leq r \leq R_{K}$, *then*

2.8 $$ \Delta_{\kappa}(K) \geq \frac{ (P_{K}-2\pi\operatorname{sn}_{\kappa}(r) )^{2}}{ \operatorname{cn}^{2}_{\kappa}(r)}, $$

*with equality if* *K* *is a geodesic disc*.

### Proof

Inequality (2.5) can be rewritten as

$$ P_{K}^{2}-A_{K}(4\pi-\kappa A_{K}) \geq P_{K}^{2} + \bigl( (2\pi-\kappa A_{K} )^{2}+\kappa P_{K}^{2} \bigr) \operatorname{sn}^{2}_{\kappa}(r) - 4\pi P_{K} \operatorname{sn}_{\kappa}(r). $$

Since $(2\pi-\kappa A_{K} )^{2}+\kappa P_{K}^{2}=4\pi^{2}+\kappa\Delta_{\kappa}(K)$, we have

$$ \bigl(1-\kappa\ \operatorname{sn}^{2}_{\kappa}(r) \bigr) \Delta_{\kappa}(K) \geq \bigl(P_{K}-2\pi\operatorname{sn}_{\kappa}(r) \bigr)^{2}. $$

Via (1.9), that is, $1-{\kappa}\cdot\operatorname{sn}^{2}_{\kappa}(r)=\operatorname {cn}^{2}_{\kappa}(r)$, then the previous inequality results in (2.8) and we complete the proof. □

### Theorem 2.2

*Let* $K\in\mathcal{C}(\mathbb{X}_{\kappa})$, *then*

$$ \begin{aligned}[b]\Delta_{\kappa}(K) \geq{}& c \bigl\{ 2\pi^{2} \bigl[\operatorname{sn}_{\kappa}(R_{K}) -\operatorname{sn}_{\kappa}(r_{K}) \bigr]^{2} +2 \bigl[P_{K}-\pi\operatorname{sn}_{\kappa}(R_{K}) -\pi\operatorname{sn}_{\kappa}(r_{K}) \bigr]^{2} \\ & -\kappa \bigl[\operatorname{sn}^{2}_{\kappa}(R_{K}) \bigl(P_{K}-2\pi\operatorname{sn}_{\kappa}(r_{K}) \bigr)^{2} + \operatorname{sn}^{2}_{\kappa}(r_{K}) \bigl(P_{K}-2\pi\operatorname{sn}_{\kappa}(R_{K}) \bigr)^{2} \bigr] \bigr\} , \end{aligned} $$

*where* $c=1/({2\operatorname{cn}^{2}_{\kappa}(R_{K}) \operatorname{cn}^{2}_{\kappa}(r_{K})})$, *and the equality holds if* *K* *is a geodesic disc*.

### Proof

Inequality (2.8) holds for $r=R_{K}$ and $r=r_{K}$, respectively:

$$\begin{gathered} \Delta_{\kappa}(K) \geq\frac{ (P_{K}-2\pi\operatorname{sn}_{\kappa}(R_{K}) )^{2}}{ \operatorname{cn}^{2}_{\kappa}(R_{K})}; \\ \Delta_{\kappa}(K) \geq \frac{ (P_{K}-2\pi\operatorname{sn}_{\kappa}(r_{K}) )^{2}}{ \operatorname{cn}^{2}_{\kappa}(r_{K})}.\end{gathered} $$

By adding the above two inequalities side by side, we have

$$\begin{aligned} \Delta_{\kappa}(K) \geq{}& c \bigl[\operatorname{cn}^{2}_{\kappa}(r_{K}) \bigl(P_{K}-2\pi\operatorname{sn}_{\kappa}(R_{K}) \bigr)^{2} +\operatorname{cn}^{2}_{\kappa}(R_{K}) \bigl(P_{K}-2\pi\operatorname{sn}_{\kappa}(r_{K}) \bigr)^{2} \bigr] \\ ={}&c \bigl[\bigl(1-\kappa\operatorname{sn}^{2}_{\kappa}(r_{K}) \bigr) \bigl(P_{K}-2\pi\operatorname{sn}_{\kappa}(R_{K}) \bigr)^{2} +\bigl(1-\kappa\operatorname{sn}^{2}_{\kappa}(R_{K}) \bigr) \bigl(P_{K}-2\pi\operatorname{sn}_{\kappa}(r_{K}) \bigr)^{2} \bigr] \\ ={}&c \bigl\{ \bigl(P_{K}-2\pi\operatorname{sn}_{\kappa}(R_{K}) \bigr)^{2} + \bigl(P_{K}-2\pi\operatorname{sn}_{\kappa}(r_{K}) \bigr)^{2} \\ & -\kappa\bigl[\operatorname{sn}^{2}_{\kappa}(R_{K}) \bigl(P_{K}-2\pi\operatorname{sn}_{\kappa}(r_{K}) \bigr)^{2} + \operatorname{sn}^{2}_{\kappa}(r_{K}) \bigl(P_{K}-2\pi\operatorname{sn}_{\kappa}(R_{K}) \bigr)^{2} \bigr] \bigr\} .\end{aligned} $$

Via elementary calculations we obtain the desired Bonnesen-style inequality. □

For $\kappa<0$, the following Bonnesen-style inequalities are immediate consequences of Theorem 2.2 with equality conditions.

### Corollary 2.1

*Let* $K\in\mathcal{C}(\mathbb{H}^{2})$, *then*

2.9 $$ \Delta_{\kappa}(K) \geq 2c \bigl\{ \pi^{2} \bigl[\operatorname{sn}_{\kappa}(R_{K}) -\operatorname{sn}_{\kappa}(r_{K}) \bigr]^{2} + \bigl[P_{K}-\pi\operatorname{sn}_{\kappa}(R_{K}) -\pi\operatorname{sn}_{\kappa}(r_{K}) \bigr]^{2} \bigr\} , $$

*where* $c=1/({2\operatorname{cn}^{2}_{\kappa}(R_{K}) \operatorname{cn}^{2}_{\kappa}(r_{K})})$, *and the equality holds if* *K* *is a hyperbolic disc*.

### Corollary 2.2

*Let* $K\in\mathcal{C}(\mathbb{H}^{2})$, *then*

2.10 $$ \Delta_{\kappa}(K) \geq 2c \bigl(P_{K}-\pi \operatorname{sn}_{\kappa}(R_{K}) -\pi\operatorname{sn}_{\kappa}(r_{K}) \bigr)^{2}, $$

*where* $c={1}/({2\operatorname{cn}^{2}_{\kappa}(R_{K}) \operatorname{cn}^{2}_{\kappa}(r_{K})})$, *with equality if and only if* *K* *is a hyperbolic disc*.

### Proof

Since

$$ \begin{gathered}\pi^{2} \bigl[\operatorname{sn}_{\kappa}(R_{K}) -\operatorname{sn}_{\kappa}(r_{K}) \bigr]^{2} + \bigl[P_{K}-\pi\operatorname{sn}_{\kappa}(R_{K}) -\pi \operatorname{sn}_{\kappa}(r_{K}) \bigr]^{2} \\ \quad\geq \bigl[P_{K}-\pi\operatorname{sn}_{\kappa}(R_{K}) -\pi\operatorname{sn}_{\kappa}(r_{K}) \bigr]^{2},\end{gathered} $$

with equality holds if and only if $R_{K} =r_{K}$, that is, *K* must be a hyperbolic disc, the Bonnesen-style inequality (2.10) follows from inequality (2.9) immediately. □

## Bonnesen-style inequalities in $\mathbb{H}^{2}$

We are seeking more Bonnesen-style inequalities in $\mathbb{H}^{2}$.

### Theorem 3.1

*Let* $K\in\mathcal{C}(\mathbb{H}^{2})$, *then*

3.1 $$ \Delta_{\kappa}(K) \geq \pi^{2} \bigl( \operatorname{tn}_{\kappa}(R_{K})-\operatorname{tn}_{\kappa }(r_{K}) \bigr)^{2} + \frac{P_{K}^{2}}{4} \biggl(\frac{1}{\operatorname{cn}_{\kappa}(r_{K})} - \frac{1}{\operatorname{cn}_{\kappa}(R_{K})} \biggr)^{2}, $$

*with equality if* *K* *is a hyperbolic disc*.

### Proof

By Theorem 2.1, $r=R_{K}$ and $r=r_{K}$ respectively lead to

$$ \Delta_{\kappa}(K) \geq \frac{ (2\pi\operatorname{sn}_{\kappa}(R_{K})-P_{K} )^{2}}{ \operatorname{cn}^{2}_{\kappa}(R_{K})} \quad\text{and} \quad\Delta _{\kappa}(K) \geq \frac{ (P_{K}-2\pi\operatorname{sn}_{\kappa}(r_{K}) )^{2}}{ \operatorname{cn}^{2}_{\kappa}(r_{K})}. $$

Adding two inequalities side by side and by the inequality $x^{2}+y^{2}\geq\frac{(x+y)^{2}}{2}$, we have

$$\begin{aligned} 2\Delta_{\kappa}(K) &\geq \frac{ (2\pi\operatorname{sn}_{\kappa}(R_{K})-P_{K} )^{2}}{ \operatorname{cn}^{2}_{\kappa}(R_{K})}+ \frac{ (P_{K}-2\pi\operatorname{sn}_{\kappa}(r_{K}) )^{2}}{ \operatorname{cn}^{2}_{\kappa}(r_{K})} \\ &\geq\frac{1}{2} \biggl\{ 2\pi \bigl(\operatorname{tn}_{\kappa}(R_{K}) -\operatorname{tn}_{\kappa}(r_{K}) \bigr)+ \biggl( \frac {P_{K}}{\operatorname{cn}_{\kappa}(r_{K})} -\frac{P_{K}}{\operatorname{cn}_{\kappa}(R_{K})} \biggr) \biggr\} ^{2}.\end{aligned} $$

For $K\in\mathcal{C}(\mathbb{H}^{2})$, $\operatorname{tn}_{\kappa}$ and $\operatorname{sn}_{\kappa}$ are respectively hyperbolic tangent $\operatorname{tanh}(x)$ and hyperbolic cosine $\operatorname{cosh}(x)$ that are strictly increasing on $[0,\infty)$. Therefore, for $r_{K}\leq R_{K}$,

$$ \operatorname{tn}_{\kappa}(R_{K})-\operatorname{tn}_{\kappa }(r_{K}) \geq 0;\qquad \frac{1}{\operatorname{cn}_{\kappa}(r_{K})}- \frac{1}{\operatorname{cn}_{\kappa}(R_{K})}\geq0. $$

By inequality $(x+y)^{2}\geq x^{2}+y^{2}$ ($x\geq0$, $y\geq0$), we have

$$ 2\Delta_{\kappa}(K) \geq2\pi^{2} \bigl(\operatorname{tn}_{\kappa}(R_{K}) -\operatorname{tn}_{\kappa}(r_{K}) \bigr)^{2}+ \frac{P_{K}^{2}}{2} \biggl(\frac{1}{\operatorname{cn}_{\kappa}(r_{K})} -\frac{1}{\operatorname{cn}_{\kappa}(R_{K})} \biggr)^{2}. $$

 □

The following Bonnesen-style inequality with equality condition for $K\in\mathcal{C}(\mathbb{H}^{2})$ is a direct consequence of Theorem 3.1.

### Corollary 3.1

*Let* $K\in\mathcal{C}(\mathbb{H}^{2})$, *then*

3.2 $$ \Delta_{\kappa}(K) \geq \pi^{2} \bigl( \operatorname{tn}_{\kappa}(R_{K})-\operatorname{tn}_{\kappa }(r_{K}) \bigr)^{2}, $$

*with equality if and only if* *K* *is a hyperbolic disc*.

### Proof

By slightly complicated elementary calculations, we have

3.3 $$ \pi^{2} \bigl(\operatorname{tn}_{\kappa}(R_{K})- \operatorname{tn}_{\kappa }(r_{K}) \bigr)^{2} + \frac{P_{K}^{2}}{4} \biggl(\frac{1}{\operatorname{cn}_{\kappa}(r_{K})} -\frac{1}{\operatorname{cn}_{\kappa}(R_{K})} \biggr)^{2}\geq \pi^{2} \bigl(\operatorname{tn}_{\kappa}(R_{K})- \operatorname{tn}_{\kappa }(r_{K}) \bigr)^{2}. $$

Then inequality (3.2) follows from (3.1) and (3.3) immediately.

Equality holds in (3.2) and (3.3) if and only if either $P_{K}=0$, which implies that *K* is a single point, or $R_{K}=r_{K}$, which means that *K* is a hyperbolic disc. □

Since

$$ \pi^{2} \bigl(\operatorname{tn}_{\kappa}(R_{K})- \operatorname{tn}_{\kappa }(r_{K}) \bigr)^{2} + \frac{P_{K}^{2}}{4} \biggl(\frac{1}{\operatorname{cn}_{\kappa}(r_{K})} -\frac{1}{\operatorname{cn}_{\kappa}(R_{K})} \biggr)^{2}\geq \frac{P_{K}^{2}}{4} \biggl(\frac{1}{\operatorname{cn}_{\kappa}(r_{K})} - \frac{1}{\operatorname{cn}_{\kappa}(R_{K})} \biggr)^{2}, $$

with equality if and only if $R_{K}=r_{K}$, which implies that *K* must be a hyperbolic disc.

Combining this inequality with inequality (3.1) immediately leads to the following Bonnesen-style inequality.

### Corollary 3.2

*Let* $K\in\mathcal{C}(\mathbb{H}^{2})$, *then*

3.4 $$ \Delta_{\kappa}(K) \geq \frac{P_{K}^{2}}{4} \biggl( \frac{1}{\operatorname{cn}_{\kappa}(r_{K})} -\frac{1}{\operatorname{cn}_{\kappa}(R_{K})} \biggr)^{2}, $$

*with equality if and only if* *K* *is a hyperbolic disc*.

## The limiting cases on the Euclidean plane $\mathbb{R}^{2}$

The limiting cases of Bonnesen-style inequalities obtained in the previous sections are known as Bonnesen-style inequalities in the Euclidean plane $\mathbb{R}^{2}$.

### Corollary 4.1

*Let* *K* *be a compact convex set in* $\mathbb{R}^{2}$. *If* $r_{K}\leq r \leq R_{K}$, *then*

4.1 $$ P_{K}^{2}-4\pi A_{K}\geq (P_{K}-2\pi r )^{2}, $$

*with equality if* *K* *is a Euclidean disc*.

### Proof

For $\kappa<0$, let $\kappa=-\frac{1}{R^{2}}$. Then inequality (2.8) becomes

$$ P_{K}^{2}-4\pi A_{K}-\frac{A_{K}^{2}}{R^{2}} \geq \frac{ (P_{K}-2\pi R\sinh(\frac{r}{R}) )^{2}}{\cosh^{2}(\frac{r}{R})}. $$

As $R\rightarrow\infty$, the inequality above leads to the following inequality by L’Hôpital’s rule:

$$ P_{K}^{2}-4\pi A_{K} \geq \lim _{R\rightarrow\infty} \biggl(P_{K}-2\pi R\sinh \biggl( \frac{r}{R} \biggr) \biggr)^{2} = (P_{K}-2\pi r )^{2}. $$

 □

### Corollary 4.2

*Let* *K* *be a compact convex set in* $\mathbb{R}^{2}$, *then*

4.2 $$ P_{K}^{2}-4\pi A_{K} \geq \pi^{2} (R_{K}-r_{K} )^{2}, $$

*with equality if and only if* *K* *is a Euclidean disc*.

### Proof

For $\kappa=-\frac{1}{R^{2}}$, inequality (3.2) becomes

$$ P_{K}^{2}-4\pi A_{K}-\frac{A_{K}^{2}}{R^{2}} \geq \pi^{2} \biggl(\frac{R\sinh(\frac{R_{K}}{R})}{\cosh(\frac{R_{K}}{R})} -\frac{R\sinh(\frac{r_{K}}{R})}{\cosh(\frac{r_{K}}{R})} \biggr)^{2}. $$

As $R\rightarrow\infty$, the inequality above becomes

$$ P_{K}^{2}-4\pi A_{K} \geq\lim _{R\rightarrow\infty} \biggl(R\sinh \biggl(\frac{R_{K}}{R} \biggr)-R\sinh \biggl(\frac {r_{K}}{R} \biggr) \biggr)^{2} = \pi^{2} (R_{K}-r_{K} )^{2}. $$

The inequality holds as an equality if and only if $R_{K}=r_{K}$, that is, *K* is a Euclidean disc. □

The limiting case of Theorem 2.2 is the following strengthening inequality of (4.2).

### Corollary 4.3

*Let* *K* *be a compact convex set in* $\mathbb{R}^{2}$. *Then*

$$ P_{K}^{2}-4\pi A_{K} \geq \pi^{2} (R_{K}-r_{K} )^{2} + (P_{K}-\pi R_{K}-\pi r_{K} )^{2}, $$

*with equality if* *K* *is a Euclidean disc*.
